# Robust sulfonated poly (ether ether ketone) nanochannels for high-performance osmotic energy conversion

**DOI:** 10.1093/nsr/nwaa057

**Published:** 2020-04-02

**Authors:** Yuanyuan Zhao, Jin Wang, Xiang-Yu Kong, Weiwen Xin, Teng Zhou, Yongchao Qian, Linsen Yang, Jinhui Pang, Lei Jiang, Liping Wen

**Affiliations:** CAS Key Laboratory of Bio-inspired Materials and Interfacial Science, Technical Institute of Physics and Chemistry, Chinese Academy of Sciences, Beijing 100190, China; University of Chinese Academy of Sciences, Beijing 100049, China; Key Laboratory of Super Engineering Plastic of Ministry of Education, Jilin University, Changchun 130012, China; CAS Key Laboratory of Bio-inspired Materials and Interfacial Science, Technical Institute of Physics and Chemistry, Chinese Academy of Sciences, Beijing 100190, China; CAS Key Laboratory of Bio-inspired Materials and Interfacial Science, Technical Institute of Physics and Chemistry, Chinese Academy of Sciences, Beijing 100190, China; School of Future Technology, University of Chinese Academy of Sciences, Beijing 100049, China; Mechanical and Electrical Engineering College, Hainan University, Haikou 570228, China; School of Science, Northwestern Polytechnical University, Xi’an 710072, China; CAS Key Laboratory of Bio-inspired Materials and Interfacial Science, Technical Institute of Physics and Chemistry, Chinese Academy of Sciences, Beijing 100190, China; School of Future Technology, University of Chinese Academy of Sciences, Beijing 100049, China; Key Laboratory of Super Engineering Plastic of Ministry of Education, Jilin University, Changchun 130012, China; CAS Key Laboratory of Bio-inspired Materials and Interfacial Science, Technical Institute of Physics and Chemistry, Chinese Academy of Sciences, Beijing 100190, China; School of Future Technology, University of Chinese Academy of Sciences, Beijing 100049, China; CAS Key Laboratory of Bio-inspired Materials and Interfacial Science, Technical Institute of Physics and Chemistry, Chinese Academy of Sciences, Beijing 100190, China; University of Chinese Academy of Sciences, Beijing 100049, China; School of Future Technology, University of Chinese Academy of Sciences, Beijing 100049, China

**Keywords:** SPEEK membrane, nanochannel, ion-selectivity, salinity gradient power generation

## Abstract

The membrane-based reverse electrodialysis (RED) technique has a fundamental role in harvesting clean and sustainable osmotic energy existing in the salinity gradient. However, the current designs of membranes cannot cope with the high output power density and robustness. Here, we construct a sulfonated poly (ether ether ketone) (SPEEK) nanochannel membrane with numerous nanochannels for a membrane-based osmotic power generator. The parallel nanochannels with high space charges show excellent cation-selectivity, which could further be improved by adjusting the length and charge density of nanochannels. Based on numerical simulation, the system with space charge shows better conductivity and selectivity than those of a surface-charged nanochannel. The output power density of our proposed membrane-based device reaches up to 5.8 W/m^2^ by mixing artificial seawater and river water. Additionally, the SPEEK membranes exhibit good mechanical properties, endowing the possibility of creating a high-endurance scale-up membrane-based generator system. We believe that this work provides useful insights into material design and fluid transport for the power generator in osmotic energy conversion.

## Teaser

The SPEEK nanochannel membrane with numerous space charges shows excellent cation-selectivity, endowing the possibility of high-performance osmotic energy conversion.

## INTRODUCTION

The ever-increasing demand for energy has pushed researchers to seek out novel and sustainable energy sources, such as salinity gradient power (SGP), also called ‘blue energy’ [1]. Osmotic power is generated when two fluidic systems with different salinities meet. In the 1970s, Loeb [2] and Norman [3] harvested osmotic energy through selective and permeable membranes. In the following decades, scientists developed a wide range of membrane-based energy capture technologies, such as pressure-retarded osmosis (PRO) [4–6] and reverse electrodialysis (RED) [7–9]. Using the RED method, SGP can be converted directly into electric current via an ion-selective membrane. However, the power density of traditional RED systems can achieve 2.2 W/m^2^ using tandem stacks, far lower than the critical value (5 W/m^2^) required for practical applications [8,10].

Inspired by electric eels, which generate voltages over 600 V using nanochannels across plasma membranes, current design principles for membranes are sourced from selectivity of nanochannels [11–14]. The nanofluidic channel system shows outstanding transport properties including excellent ion-selectivity and high ionic throughput from surface charge effects in the intense nanoconfinement, which can be used in osmotic energy conversion [15–18]. A wide range of materials has been developed following these principles, including inorganic materials (e.g. single-layer MoS_2_ nanopore membrane [19] and boron nitride nanotube [20]), organic membranes (e.g. polymeric carbon nitride membrane [21], PET membrane with poly-L-lysine [22]), composite membranes (e.g. MXene/ANF [23] and SNF/AAO composite membranes [24]) and soft matter (e.g. hydrogel [25]). Although these works have made progress in fundamental study and provide theoretical guidance for selective membranes in osmotic power conversion, the advance of materials is hindered by complicated preparation and inadequate mechanical properties, limiting large-scale use of SGP generation [22,26,27]. Therefore, there is a need to construct membranes with desirable performance and facile synthesis.

Herein, we demonstrate sulfonated poly (ether ether ketone) (SPEEK) membranes with numerous nanochannels, created using a nonsolvent induced phase separation (NIPS) method, for converting SGP to electric energy. The negatively charged membrane contains a spongy porous supporting layer and a dense skin layer, which is rich in parallel continuous nanochannels. The nanochannels dominate ion-selective transport by facilitating transport of cations and rejecting anions [28,29]. As a result, the output power density of the membrane-based device reaches the commercialization benchmark of 5 W/m^2^. Additionally, the power density can be improved by adjusting the membrane thickness and sulfonation degree (DS). Furthermore, SPEEK membranes demonstrate outstanding mechanical property and high endurance, with the potential for practical industrial uses [30–32]. This work provides inspiration for manipulating cation-selective nanochannels and accelerating the development of a membrane-based osmotic power generator.

## RESULTS AND DISCUSSION

**Figure 1.**
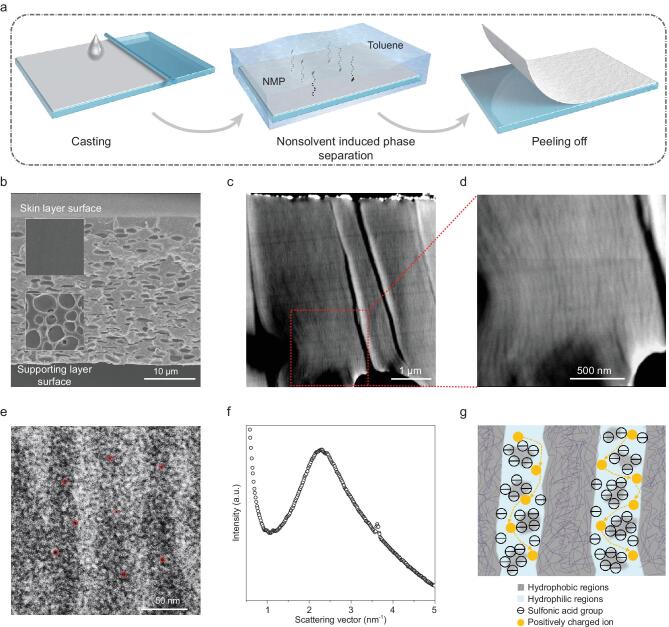
Fabrication method of the SPEEK membrane and characterization of nanochannels. (a) Steps for preparing membranes based on NIPS process. (b) Asymmetric morphology of SPEEK membrane cross-section, consisting of the thin skin layer and supporting layer. Insets: scanning electron microscopy (SEM) views of skin layer surface and the supporting layer surface. (c) Cross-section of the skin layer obtained from HAADF-STEM. (d) Magnified view of (c). Numerous hydrophilic nanochannels are found through the skin layer with sizes ranging from 20 to 60 nm. (e) TEM images of the SPEEK membrane skin layer at magnification. The dark spots in the image represent the ionic clusters, which are marked with red circles as examples, with size ranging from 1.5 to 6 nm. (f) SAXS data of the SPEEK membrane. (g) Schematic of continuous ion transport in the upright oriented channels. The negatively charged ionic clusters aggregate and are seen as space charges in the nanochannels.

The NIPS method was employed to fabricate the SPEEK membranes (Fig. 1a). A series of membranes with different treatments was prepared (Supplementary Table 1 and Fig. 1), with the free-standing membranes showing robust performance in dry and wet states (Supplementary Fig. 2). The proton nuclear magnetic resonance (^1^H-NMR), Fourier transform infrared (FT-IR) and gel permeation chromatography (GPC) measurements were conducted to confirm the structure and molecular weight (Supplementary Figs 3–6). The SPEEK membrane has an asymmetric structure, with a thin skin layer and sponge-like supporting layer (Fig. 1b and Supplementary Fig. 1). The skin layer has an extraordinarily smooth surface, and the supporting layer exhibits abundant interconnected pores (Fig. 1b, insets; Supplementary Fig. 7). This asymmetric structure can be attributed to use of the NIPS method. Liquid-Liquid (L-L) phase separation occurred preferentially at the contact surface of casting solution (N-methyl-2-pyrrolidinone (NMP) solution of SPEEK) and nonsolvent (toluene). As a result, solid precipitated firstly at the top surface of the casting solution, formatting the skin layer of the SPEEK membrane. During the process, the exchange of NMP and toluene is fast, so the skin layer is very thin and compact. Then the skin layer impeded further occurrence of L-L phase, causing a low exchange rate of casting solution and nonsolvent. Thus, the supporting layer was relatively thick and full of sponge-like pores. Water contact angle was also measured to monitor the different wettability of two-faced SPEEK membranes (Supplementary Fig. 8), with results of ∼72° on the skin layer surface and ∼84° on the supporting layer surface because of the different roughness of the two sides (Supplementary Fig. 7).

To further investigate the morphology of the skin layer, we employed transmission electron microscopy (TEM) for the microscopic structures (Fig. 1c–e). High angle annular dark field scanning transmission electron microscopy (HAADF-STEM) imaging mode (Fig. 1c and d) provides a contrast strongly dependent on the atomic number (∼Z^2^), and thus the stained polymer phase which is hydrophilic looks much brighter in images. The numerous stripe-like nanochannels are aligned throughout the skin layer and perpendicular to the membrane surface (Fig. 1c). The nanochannels have sizes ranging from 20 to 60 nm in the magnified view in Fig. 1d. TEM was employed to further obtain the morphology, as shown in Fig. 1e, in which the dark and light regions are related to hydrophilic and hydrophobic domains. Relatively continuous nanochannels are formed by ionic clusters (∼2–6 nm), which is beneficial for the movement of ions [33,34]. The size of ionic clusters (2.7 nm) can be confirmed by small-angle X-ray scattering (SAXS) (Fig. 1f), and is calculated by d = 2π/q [33] where q (scattering vector) is 2.3 nm^−1^. As shown in the scheme of Fig. 1g, each ionic cluster is full of negative charges (sulfonic acid groups) and is seen as the space charge. Numerous space charges aggregate and form hydrophilic nanochannels. The connectivity of the nanochannels is beneficial to the selective transportation and conductivity of ions. Nanochannels are similar to continuous perpendicular xylem vessel elements, which transport water and minerals from the bottom to the top of plants [35,36].

**Figure 2.**
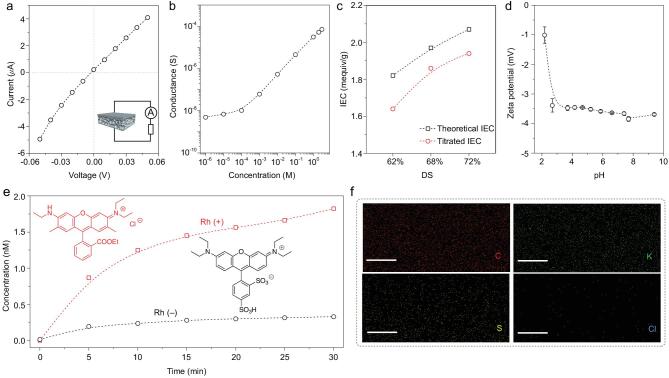
Ionic transport property and selectivity of the SPEEK membrane. (a) *I-V* response of the SPEEK membrane recorded in 0.1 M KCl solution. (b) Ionic conductance of the SPEEK membrane as a function of the electrolyte concentrations. (c) IEC of SPEEK membranes with different DS. (d) Zeta potential of the SPEEK membrane at pH from 2.00 to 10.00 in 1 mM KCl. (e) Time-concentration curves of the permeation experiments using two oppositely charged dyes: Rh (+) (red square) and Rh (−) (black circle). The permeability rate of the positively charged Rh (+) is much larger than that of the negatively charged Rh (−) because of the good cation-selectivity of the SPEEK membrane. (f) EDX element mapping for the cross-section of the skin layer after immersion in KCl solution. Scale bar: 500 nm. The SPEEK membrane contains large amounts of carbon and sulfur. As the SPEEK membrane is negatively charged because of the sulfate groups, it mainly absorbs potassium ions, yet expels chloride ions.

The ionic transport behaviors of the SPEEK membrane were characterized by measuring the transmembrane ionic current in an electrochemical system with Ag/AgCl electrodes (Supplementary Fig. 9). Under a series of applied voltages in 0.1 M KCl electrolyte solution, the asymmetric ion transport with a mild rectification ratio was recorded (Fig. 2a). Figure 2b indicates that the transmembrane conductance deviates from bulk behavior by 0.1 mM [37], indicating that the ion transport through the SPEEK membrane is fully charge-governed [38]. The conductivity of the SPEEK membrane displays dependence on salt concentration. With the decline of KCl solution concentration, the thickness of the electric double layer increases and eventually extends well with the nanochannel. The ion concentration inside the channel is determined by the charge on the ion cluster rather than the bulk concentration.

The potential and current are determined by discrepancies in diffusion rate of positive and negative charges. Thus, ion-selectivity plays a vital role in generation of SGP. Firstly, ion exchange capacity (IEC) shows the number of sulfonic acid groups of SPEEK membrane and is significant for cation-selectivity. Thus, we obtained theoretical IEC and titrated IEC values of SPEEK membranes with different DS via the results of ^1^H NMR and an acid-base back-titration method. As shown in Fig. 2c, the IEC increases with increasing DS. Additionally, Fig. 2d shows that the zeta potential of SPEEK membrane surface is negatively charged. To further verify the cation-selectivity of the SPEEK membrane, two fluorophores with high quantum yields and opposite charges, rhodamine 6G (Rh (+)) and sulforhodamine (Rh (−)), were used in tests, both with characteristic fluorescence emission spectra. The permeability rate of Rh (+) was larger than that of Rh (−), proving that the SPEEK membranes have excellent cation-selectivity (Fig. 2e). In addition, energy dispersive X-ray spectroscopy (EDX) element mapping was conducted on a cross-section of the SPEEK membrane to confirm the ionic selectivity. The membrane was immersed in 0.1 M KCl solution for 1 h before mapping. As shown in Fig. 2f, the SPEEK membrane contains large amounts of carbon and sulfur. The content of potassium in membrane is much higher than that of chlorine, suggesting cation-selectivity in agreement with the result of permeation experiment. Thus, SPEEK membranes possess extraordinary cation-selectivity, which is favorable to osmotic power conversion.

**Figure 3.**
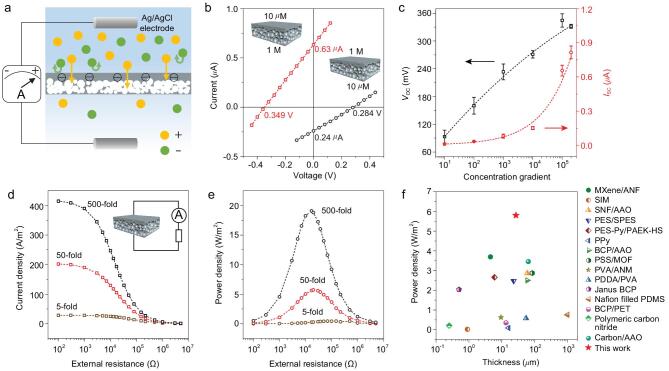
Osmotic energy harvesting performance of the SPEEK membrane-based generator. (a) Experimental set-up containing salt solutions with different concentrations separated by a SPEEK membrane. An ionic flux driven by chemical potential through the membrane is screened by the negatively charged membrane, producing a diffusion current composed of mostly cations. (b) *I-V* curves of the SPEEK membrane under two different salinity gradient configurations. (c) *V*_OC_ and *I*_SC_ are measured in a series of concentration gradients. As the concentration gradient increases, both *V*_OC_ and *I*_SC_ gradually increase. The low-concentration (KCl) solution is placed at the skin layer surface side and is fixed at 10 *μ*M. Current density (d) and output power density (e) of SPEEK membrane as functions of load resistance under three salinity gradients. The harvested electrical power can be transferred to an external circuit to supply an external load resistance. The low-salinity solution is placed at the skin layer side and fixed at 0.01 M. High-salinity solution is tunable from 0.05 to 5 M. (f) Power generation performance of SPEEK membranes (red star) compared with state-of-the-art osmotic power generators [21,23,24,39–50].

Next, we applied a negatively charged SPEEK membrane-based generator to convert SGP into electricity. When two solutions of different concentrations meet on either side of SPEEK membrane, a chemical potential gradient is generated and ions are driven across the membrane, producing an ionic diffusion flux towards the equilibrium state (Fig. 3a) [19]. Cations diffuse spontaneously through the ion-selective membrane according to their charge polarity, while the counter-ions are mostly repelled, resulting in a net charge migration of ions. The Gibbs free energy of mixing can be partially extracted from reactions on electrodes in the solutions [10].

To investigate the potentials in salt gradient, we recorded *I-V* curves under a series of KCl concentration gradients through the SPEEK membrane. The short-circuit current (*I*_SC_) and open-circuit voltage (*V*_OC_) are obtained from the intercepts of the current and voltage axes, respectively. Note that *V*_OC_ includes two parts [51], the diffusion potential (*E*_diff_) and the redox potential (*E*_redox_) (Supplementary Fig. 10). *E*_diff_ results from differences in the diffusive fluxes of cations and anions. *E*_redox_ is produced by the non-equal potential drop at the electrode-solution interfaces, which follows the Nernst equation [19,21]. As shown in Fig. 3b, when 1 M KCl solution was put on the supporting layer side and 10 *μ*M KCl solution was set on the other side, the *I*_SC_ and *V*_OC_ were approximately 0.63 *μ*A and 349 mV, respectively. However, by switching the electrolytes, the *I*_SC_ decreased to about 0.24 *μ*A and the *V*_OC_ decreased to 284 mV. Thus, the predominant transport direction for cations is from the supporting layer side to the skin layer side. Here, the asymmetric structure provides ion-storage and ion-selective functions, in which the pores in the supporting layer store a large number of ions and the nanochannels in the skin layer perform ion screening [24]. Thus, the following measurements were all performed using the preponderant configuration. Figure 3c shows the growth of *V*_OC_ and *I*_SC_ as the concentration gradient increases. We calculated *E*_diff_, *E*_redox_, the transference number (*t*_+_) and the energy conversion efficiency (*η*) under a series of concentration gradients [51] (Supplementary Tables 2 and 3). The *η* decreases as the concentration gradient increases from 10-fold to 2 × 10^5^-fold. It is worth noting that more concentrated solutions result in more severe concentration polarization phenomena at the membrane-solution interface, which significantly reduces the practical power output [52]. Moreover, the values of *t*_+_ at different salinity gradients are > 0.5, confirming the above-mentioned outstanding cation-selectivity.

The harvested electric power can be conveyed to an external circuit with an electrical load resistor (*R*_L_, inset in Fig. 3d). Under different salinity gradients, we probed the relationship between the output power density and external load resistance. The power density of the resistor in the circuit is calculated using the equation: *P*_output_ = *I*^2^ × *R*_L_. A low salinity solution (0.01 M NaCl) was placed on the skin layer side, and a high concentration solution on the other side varying from 0.05 M to 5 M. The current density gradually decreases along with the growth of *R*_L_ (Fig. 3d) and the output power density reaches a maximum at the load resistance, which is equal to the internal resistance of the membrane (Fig. 3e). Additionally, the SPEEK membrane-based generator revealed high performance in osmotic energy conversion. As salinity gradients increase, the output power increased from 0.4 W/m^2^ (5-fold) to 20.2 W/m^2^ (500-fold), suggesting the possibility of harvesting osmotic energy where the salt-lake meets the river. In addition, it is significant for the membrane to adapt to a wide pH range as the pH values of sea water and river water vary in different areas. As shown in Supplementary Fig. 11, when a SPEEK membrane with a DS of 62% was placed in 0.01 M/0.5 M NaCl solution, the excellent osmotic power conversion performance was maintained in the electrolyte from pH 3 to 11. As shown in Fig. 3f, comparing other membrane-based power generators, the output power density of the SPEEK membrane in this work is higher than the performances of state-of-the-art osmotic power generators.

**Figure 4.**
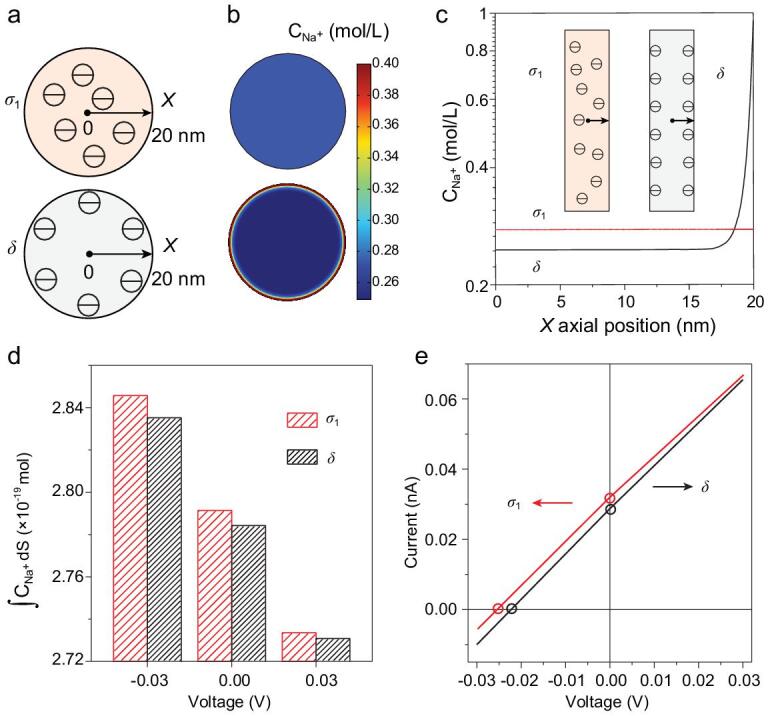
(a) Schematic of nanochannel cross-sections with σ_1_ and δ, respectively. The *X* = 0 nm position refers to the center of the channel cross-section and the *X* = 20 nm position refers to the channel wall. (b, c) Numerical simulation results of the Na^+^ concentration distribution along the position (*X*) changing. The bulk NaCl solution with concentration gradient set at 0.01 M/0.5 M. (d) Integration of Na^+^ concentration in nanochannels with space charge and surface charge when the applied biases are set to −0.03 V, 0 and 0.03 V, demonstrating that a higher concentration of Na^+^ exists in space-charged nanochannels, which facilitates ion conductivity. (e) *I-V* curves of the nanochannel with space charge and surface charge, showing different *I*_SC_ and *V*_OC_.

Constructing nanofluidic channels with space charge has been considered to improve the performance of power output [23]. Compared with membranes with nanochannels with surface charges, the SPEEK membrane rich in nanochannels with space charges shows relatively higher output power density. To provide further understanding of the relationship between the form of charge distribution in nanochannels and ion transportation behaviors, continuum theoretical mathematics models were constructed using finite elements based on Poisson and Nernst-Planck (PNP) equations. The fluidic transport route inside the skin layer was simplified to be a single nanochannel (Supplementary Figs 12 and 13). We explored the difference between two types of charge distribution (Fig. 4a), space charge (σ_1_) and surface charge (δ), assuming the total charge is equal in a nanochannel. To make the simulation more realistic, the salinity gradient (0.01 M/0.5 M NaCl) was employed. The calculated sodium ion concentration profile of nanochannels with space charges is homogeneous from the center to the wall of the channel, as shown in Fig. 4b and c, whereas that of the surface-charged nanochannels mainly distributes on the wall of channel. When the applied voltages are set at −0.03 V, 0 and 0.03 V, integration of Na^+^ concentration in nanochannels with space charges is higher than that of surface-charged nanochannels (Fig. 4d), providing better selectivity and promoting ion conductivity. As seen in Fig. 4e, the predicted *I-V* curves show that nanochannels with space charges have higher *I*_SC_ and *V*_OC_, implying better osmotic power conversion properties. Corresponding simulated ion transport behaviors without salt gradient using KCl solution were conducted, and also showed better selectivity and conductivity of space-charged nanochannels (Supplementary Fig. 14a and b).

**Figure 5.**
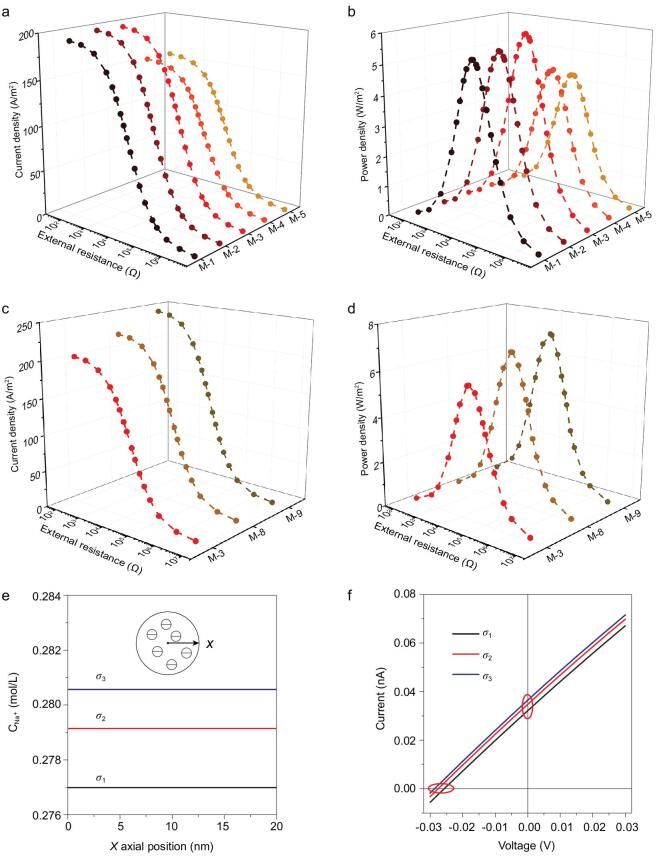
Ultrahigh output power density of SPEEK membrane-based osmotic power generators. Current density (a) and output power density (b) of membranes with different thickness as functions of load resistance. When the load resistance increases, the current density decreases, and the output power reaches a maximum at a moderate resistance. Current density (c) and output power density (d) of membranes with different DS as functions of load resistance. The output power density increases along with increasing DS. (e) Under a salt gradient of 0.01 M/0.5 M, numerical simulation results of the Na^+^ concentration distribution along the radial position (*X*) in space-charged (σ_1_, σ_2_ and σ_3_) nanochannels, which correspond to different DS (62%, 68% and 72%). (f) *I-V* curves of the nanochannel with three space charges, showing different *I*_SC_ (Y-axis intercept) and *V*_OC_ (X-axis intercept) and confirming that high space charge leads to better SGP performance.

The selectivity of the SPEEK membrane can be strengthened by controlling the length and charge density of nanochannels in the skin layer [49]. By changing the gap between the substrate and the scraper in the process of membrane preparation, we obtained a series of membranes with different thicknesses (M-1 to M-5, Supplementary Table 1 and Supplementary Fig. 1). Meanwhile, adjusting the mass fraction of SPEEK solution can also regulate the length of nanochannels (M-3, M-6 and M-7, Supplementary Table 1). The current densities and output power densities were tested in asymmetric NaCl solutions (0.01 M/0.5 M). In Fig. 5a and b, as well as Supplementary Fig. 15a–c, with the growth of membrane thickness, osmotic current density and the power density are shown to reach maximum when applying M-3. This is mainly because nanochannels of short length have insufficient ion-selectivity and strong ion concentration polarization. In addition, the membrane resistance increases when the thickness of membranes is higher than 27 *μ*m. It should be noted that the amount of charge density for the nanochannels also affects the membrane selectivity [23]. The power densities of membranes with different DS (M-3, M-8 and M-9, Supplementary Table 1) were tested in a salinity gradient of 50-fold (0.01 M/0.5 M). As shown in Fig. 5c and d, as well as Supplementary Fig. 15d, the power density increases with increases in DS. Notably, increasing DS results in absorption of plenty of water, remarkably enhancing the ion transport through an increase in ionic conductance [53]. Therefore, such continuous promotions for ion transport facilitate osmotic power conversion performance.

Further simulation-based PNP theory was carried out to obtain further insight into the relationship between space charge density and ion transport (Supplementary Section 8). In Fig. 5e and f, similar to the above simulation, we illustrate that cation concentration increases with the growth of space charge density, implying higher selectivity and conductivity. Higher *I*_SC_ and *V*_OC_ from higher charge density display better osmotic power conversion properties, which agrees well with the experimental results that the higher DS of SPEEK membrane leads to higher output power density. We also predict the corresponding simulated ion transport behaviors without salt gradient in KCl solution, displaying better conductivity of nanochannels with higher space charge density (Supplementary Fig. 14c and d).

**Figure 6.**
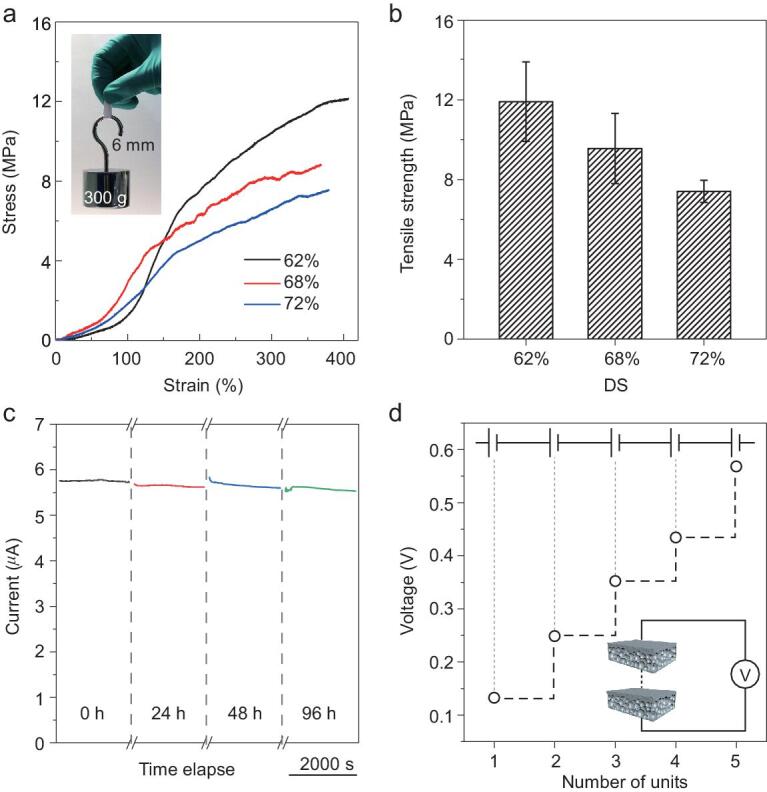
Stability and robustness of a high-performance SPEEK membrane-based power generator. (a) Tensile stress-strain curves of the SPEEK membranes with different DS. Insets: a membrane (M-3, DS of 62%) of 0.03 mm thickness and 6 mm width can endure 300 g of weight. (b) Strength of SPEEK membranes with different DS. (c) Stability tests of the membrane-based energy device in mixing artificial river water and artificial seawater. (d) An individual membrane-based cell can provide approximately 120 mV, and the output voltages show a linear relationship with the number of units under 50-fold concentration gradient.

Mechanical performance of membranes is crucial to large-scale practical applications for membrane-based power generators. As illuminated in Fig. 6a and b, the SPEEK membranes demonstrate good mechanical performance, sufficiently tough and ductile for large-scale practical applications. Besides, there is a decrease in the tensile strength with increasing DS. When SPEEK membranes are exposed to water, the amorphous structure absorbs the liquid and swells. The incorporation of a sulfonic group into a polymer disrupts the intermolecular forces between the macromolecules. Additionally, the continuous outputs of diffusion current stayed still for 96 h by mixing artificial seawater and artificial river water (Fig. 6c), which suggests admirable stability and high endurance of SPEEK membrane-based energy devices. A generator based on a SPEEK membrane with robustness and long-term persistent properties has great possibilities in practical fields. Besides, in Fig. 6d, the output voltages display a linear relationship of about 120 mV per unit cell when the salinity concentration gradient is 50-fold. Tandem SPEEK membrane-based power generator stacks can generate high voltages and demonstrate great potential for supplying electronic devices, ensuring that the application of membrane-based generators is achievable.

We confirmed the outstanding oxidative stability of the SPEEK membrane with a DS of 62% by soaking the membrane in Fenton's reagent (3% H_2_O_2_ containing 2 ppm FeSO_4_) at 80°C for 1 h, with remaining weight of about 96.7%. For practical applications, the SPEEK membranes have the potential to retain good chemical stability when exposed to the environment. The high-temperature thermal stability of the membrane was confirmed in thermogravimetric analysis (TGA) curves (Supplementary Fig. 16). Below 200°C, the weight loss was associated with loss of residual solvent and dehydration, that is, evaporation of free water and bound water. It can be deduced that the SPEEK membrane would have good SGP conversion performance within wide ranges of pH and temperature, because of its great chemical and thermal stability.

## CONCLUSION

In summary, we fabricated an ion-selective SPEEK membrane containing numerous nanochannels via NIPS method for harvesting SGP. Benefiting from an anionic sulfonic group in the skin layer, the negatively charged nanochannel membrane shows remarkable and stable cation-selectivity and good ion conductivity. The SPEEK membrane has been applied to osmotic energy conversion, and the output power density of the device can achieve ∼5.8 W/m^2^ with a salinity gradient of 0.5 M/0.01 M. Additionally, the SPEEK membrane exhibits prominent stability and outstanding mechanical performance, prerequisites for industrial applications in SGP generation. It is anticipated that this system with its asymmetric and highly selective membrane will be used in advances in the fields of energy conversion, water treatment and desalination.

## METHODS

### Materials

PEEK was produced by Changchun Jilin University Special Plastic Engineering Research Co., Ltd. (China). NMP,  sulfuric acid (98 wt%), n-hexane and toluene were purchased from Sinopharm Chemical Reagent Co., Ltd. (China). Other chemicals were all analytical grade and acquired commercially.

### Synthesis of SPEEK

PEEK (50 g) and sulfuric acid (98 wt%, 750 ml) were put into three-necked flasks with stirring at a temperature of 50°C for 8.5 h, 10.5 h and 12.5 h, respectively, forming brown solutions. Subsequently, the brown solutions were poured into ice-water and light pink polymers were produced. The polymers were washed with purified water several times until the pH of the mixtures turned to 7, then dried at 60°C. The resulting products were SPEEK and the DS of the products were 62%, 68% and 72%, respectively, as calculated by NMR.

### Membrane fabrication

As shown in Fig. 1a, a delayed phase separation process was employed to prepare the SPEEK membranes. In detail, 2 g SPEEK was dissolved in 8 g NMP, forming a homogeneous sticking solution (20 wt%), and then rested for 24 h to remove the air bubbles. Subsequently, the solution was cast onto a spotless glass substrate using a pre-cleaned scraper as casting machine. The thickness of the SPEEK membranes was adjusted from 6 to 80 μm by changing the gap between the substrate and the scraper. Then, the substrate was transferred to a toluene bath for 4 days. After that, the membrane was peeled off the glass substrate followed by immersion in n-hexane and a water bath for several seconds, respectively. Finally, the membranes were dried naturally at room temperature. As a comparison, SPEEK solutions with mass fractions of 15% and 25% were also used to prepare SPEEK membranes using the same method.

### Electrical measurements

As shown in Supplementary Fig. 9, the measurement setup was built to investigate the ionic transport property and the osmotic power density of the membranes. The ionic current through the membrane was measured with a Keithley 6487 picoammeter (Keithley Instruments, Cleveland, OH). The membrane was fixed between a two-compartment cell and the transmembrane potential was obtained using a pair of homemade Ag/AgCl electrodes, which were put at two different sides of the cell. The testing membrane area was about 3 × 10^4^*μ*m^2^.

### Characterizations

^1^H-NMR spectra were recorded with the Bruker Advance III (400 MHz, Karlsruhe, Baden-Wurttemberg, Germany) with DMSO-d_6_ as the solvent and tetramethylsilane (TMS) as the internal standard.

FT-IR spectra were recorded with a Varian FT-IR spectra meter (Excalibur 3100).

GPC PLGPC 220 eluted by N,N-dimethylformamide (DMF) with 0.01 M LiCl at 80°C was employed to determine the molecular weight of SPEEK.

SEM (SU4800, HITACHI Instrument Co., Germany) was applied to obtain two-surface morphology and cross-section morphology of the membrane with an acceleration voltage of 5 kV. EDX was employed to map the element distribution on the cross-section of the membrane with SEM.

Prior to TEM, the samples were first treated with a 0.5 M lead acetate solution in 50°C for 24 h and then washed with deionized water to remove the lead ions in the membrane. The membrane sample was then dried. It was further fixed in epoxy before being cut into thin slices (thickness: 80 nm) using the Ultramicrotome (Leica EM UC7). In Fig. 1c and d, the membrane morphology was studied by TEM using a JEM-2100 microscope in HAADF view operated at 200 kV. As shown in Fig. 1e, the morphology of the SPEEK membrane skin layer was captured by TEM (HITACHI H-7650) with an acceleration voltage of 80 kV.

The SAXS data were collected with a SAXS instrument (SAXSpace, AntonPaar). The SAXS data of all the membranes were collected in the dry state after pre-treatment by soaking in a 2 M CsCl solution for 24 h, washing with water, and drying in an oven at 90°C for 24 h.

IEC was determined by an acid-base back-titration method using phenolphthalein as an indicator. The membrane was immersed in saturated NaCl aqueous solution for 48 h to ensure that H^+^ ions were replaced by Na^+^ ions. NaOH solution (0. 1 M) was employed to titrate this solution. The IEC values were calculated from the equation as follows:

$$\begin{eqnarray*}
\text{IEC}({\rm{mequiv}}/{{\rm g}}) = \frac{{0.1 \times 1000 \times {\text{V}_{\text{NaOH}}}}}{{{\text{W}_{\text{dry}}}}},\end{eqnarray*}$$

where the ${\text{V}_{\text{NaOH}}}$ is the titrated volume of NaOH solution, ${\text{W}_{\text{dry}}}$ is the dry weight of the membrane in H^+^ form.

Surface zeta potential was conducted on the SurPASSTM 3 Zeta Potential Analyzer.

Permeation experiments were performed using a two-cell system separated by the membrane. The permeability rate was tested separately to avoid interference. The supporting layer side was facing the feed solution (0.1 mM). The permeability was monitored using a spectrofluorophotometer (RF-5301 PC, SHIMADZU, Japan).

A Shimadzu AG-I 20-kN Universal Tester (SHIMADZU, Japan) was employed to evaluate the mechanical properties of the SPEEK membranes with a strain rate of 2 mm/min. The membranes were immersed in purified water for 3 days before tensile test.

### Numerical simulation (axial symmetry)

The influence of space charge and surface charge in nanochannels was theoretically investigated using a commercial finite-element software package COMSOL Multiphysics [23]. When the system reaches a stationary regime, the ionic flux should satisfy the steady state. The couple equations were solved neglecting hydrodynamic effects and assuming appropriate boundary conditions. A sketch of the computation domain is shown in Supplementary Fig. S13. To gain an affordable computation scale, the fluidic pathway through the membrane was simplified to be a 1000-nm long single channel (diameter: 40 nm). Two electrolyte reservoirs (400 × 200 nm) were added to minimize the influence of the resistance of mass transfer at the entrance and exit. In this work, the surface charge and space charge were set according to the calculation, within the proper ranges [23] (see details in Section 8 in Supplementary Data).

## Supplementary Material

nwaa057_Supplemental_File
