# Corrigendum to Robust sulfonated poly (ether ether ketone) nanochannels for high-performance osmotic energy conversion

**DOI:** 10.1093/nsr/nwaa217

**Published:** 2020-09-17

**Authors:** Yuanyuan Zhao, Jin Wang, Xiang-Yu Kong, Weiwen Xin, Teng Zhou, Yongchao Qian, Linsen Yang, Jinhui Pang, Lei Jiang, Liping Wen

**Affiliations:** CAS Key Laboratory of Bio-inspired Materials and Interfacial Science, Technical Institute of Physics and Chemistry, Chinese Academy of Sciences, Beijing 100190, China; University of Chinese Academy of Sciences, Beijing 100049, China; Key Laboratory of Super Engineering Plastic of Ministry of Education, Jilin University, Changchun 130012, China; CAS Key Laboratory of Bio-inspired Materials and Interfacial Science, Technical Institute of Physics and Chemistry, Chinese Academy of Sciences, Beijing 100190, China; CAS Key Laboratory of Bio-inspired Materials and Interfacial Science, Technical Institute of Physics and Chemistry, Chinese Academy of Sciences, Beijing 100190, China; School of Future Technology, University of Chinese Academy of Sciences, Beijing 100049, China; Mechanical and Electrical Engineering College, Hainan University, Haikou 570228, China; School of Science, Northwestern Polytechnical University, Xi’an 710072, China; CAS Key Laboratory of Bio-inspired Materials and Interfacial Science, Technical Institute of Physics and Chemistry, Chinese Academy of Sciences, Beijing 100190, China; School of Future Technology, University of Chinese Academy of Sciences, Beijing 100049, China; Key Laboratory of Super Engineering Plastic of Ministry of Education, Jilin University, Changchun 130012, China; CAS Key Laboratory of Bio-inspired Materials and Interfacial Science, Technical Institute of Physics and Chemistry, Chinese Academy of Sciences, Beijing 100190, China; School of Future Technology, University of Chinese Academy of Sciences, Beijing 100049, China; CAS Key Laboratory of Bio-inspired Materials and Interfacial Science, Technical Institute of Physics and Chemistry, Chinese Academy of Sciences, Beijing 100190, China; University of Chinese Academy of Sciences, Beijing 100049, China; School of Future Technology, University of Chinese Academy of Sciences, Beijing 100049, China

Yuanyuan Zhao and Jin Wang are equally contributed to ‘Robust sulfonated poly (ether ether ketone) nanochannels for high-performance osmotic energy conversion’ (*National Science Review*, Volume 7, Issue 8, 2020, Pages 1349–1359, doi: 10.1093/nsr/nwaa057). In the original published version of this manuscript, only Yuanyuan Zhao was listed as first author. The published manuscript has now been corrected to also include Jin Wang as a first author.

