# Supplementary material for: Robust sulfonated poly (ether ether ketone) nanochannels for high-performance osmotic energy conversion
Source: Natl Sci Rev. 2020 Apr 2;7(8):1349–59. doi: 10.1093/nsr/nwaa057 (PMC8288931; doi:10.1093/nsr/nwaa057)
Supplement: nwaa057_Supplemental_File [file nwaa057_supplemental_file.doc]

*Supplementary materials*

**Robust Sulfonated Poly (Ether Ether Ketone) Nanochannels for High-performance Osmotic Energy Conversion**

*Yuanyuan Zhao^1,2^, Jin Wang^3^,* *Xiang-Yu Kong^1^, Weiwen Xin^1,4^, Teng Zhou^5^,* *Yongchao Qian^6^, Linsen Yang^1,4^, Jinhui Pang^3^*, Lei Jiang^1,4^, Liping Wen^1,4^**

1 CAS Key Laboratory of Bio-inspired Materials and Interfacial Science, Technical Institute of Physics and Chemistry, Chinese Academy of Sciences, Beijing 100190, P. R. China

2 University of Chinese Academy of Sciences, Beijing 100049, P. R. China

3 Key Laboratory of Super Engineering Plastic of Ministry of Education, Jilin University, Changchun 130012, P. R. China

4 School of Future Technology, University of Chinese Academy of Sciences, Beijing 100049, P. R. China

5 Mechanical and Electrical Engineering College, Hainan University, Haikou, Hainan 570228, P. R. China

6 School of Science, Northwestern Polytechnical University, Xi’an 710072, P. R. China

This PDF file includes:

Section 1. SPEEK membranes with different treatments

Section 2. Characterization of the SPEEK membrane

Section 3. Electrical measurements

Section 4. Ion selectivity of the membrane

Section 5. Electrode calibration

Section 6. Energy conversion efficiency

Section 7. The stability of generator in solutions at different pH

Section 8. Numerical simulation

Section 9. Output power densities of different SPEEK membranes

Section 10. The thermal property

Section 1. SPEEK membranes with different treatments (M1-M9)

Table 1. Specifications of the investigated SPEEK membranes.

| Membrane | Polymer concentration | Sulfonation degree (DS) | Thickness (*μ*m) |
| --- | --- | --- | --- |
| M-1 | 20% | 62% | 6 |
| M-2 | 20% | 62% | 13 |
| M-3 | 20% | 62% | 27 |
| M-4 | 20% | 62% | 55 |
| M-5 | 20% | 62% | 68 |
| M-6 | 15% | 62% | 20 |
| M-7 | 25% | 62% | 33 |
| M-8 | 20% | 68% | 32 |
| M-9 | 20% | 72% | 26 |





Figure 1. The cross section of the SPEEK membranes with different thickness.

Section 2. Characterization of the SPEEK membrane

2.1 The optical photograph of the SPEEK membrane


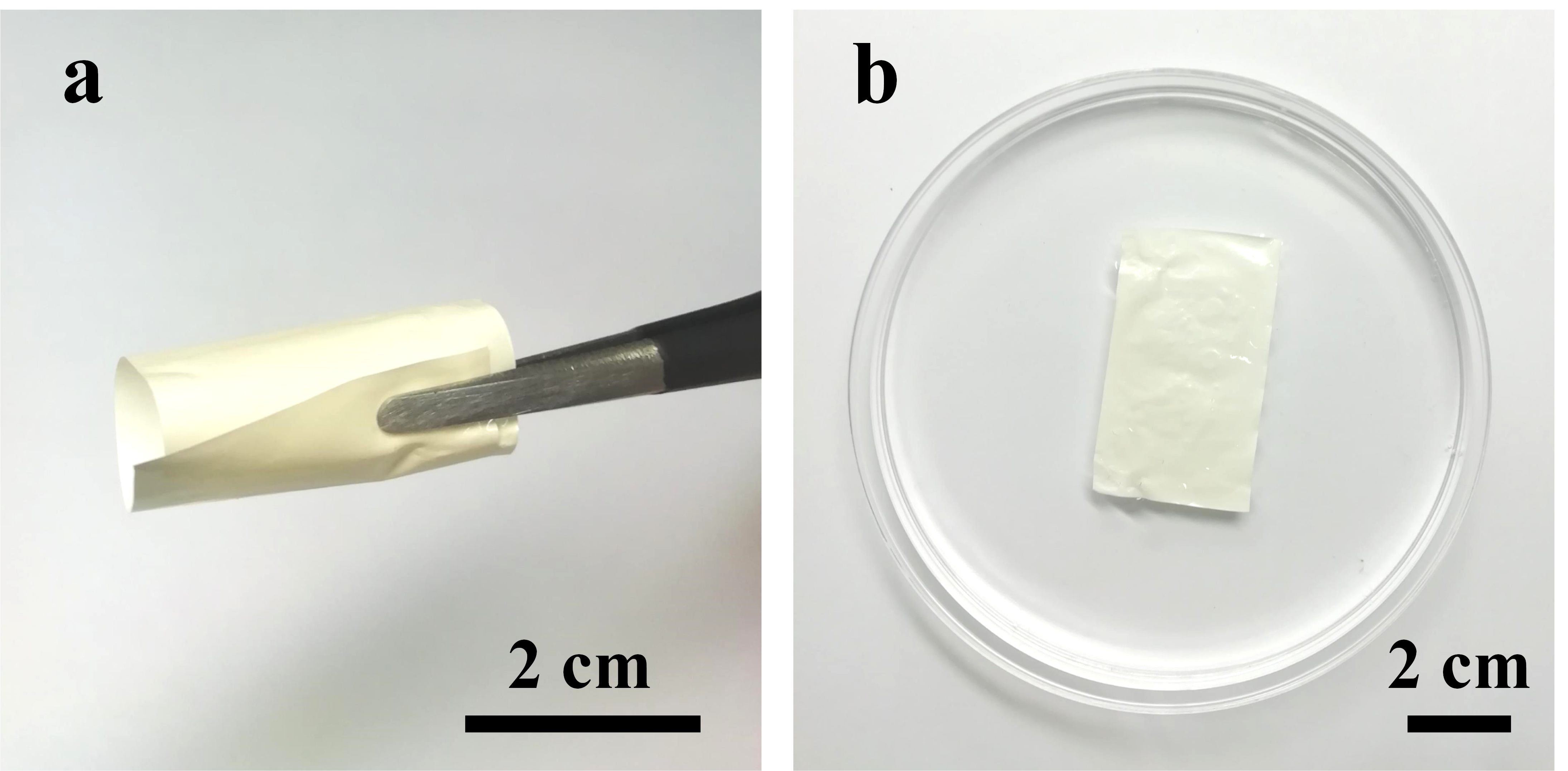


Figure 2. Digital photo of the SPEEK membrane with an approximate thickness of 27 *μ*m in (a) dry state and (b) wet state.

2.2 ^1^H-NMR measurements


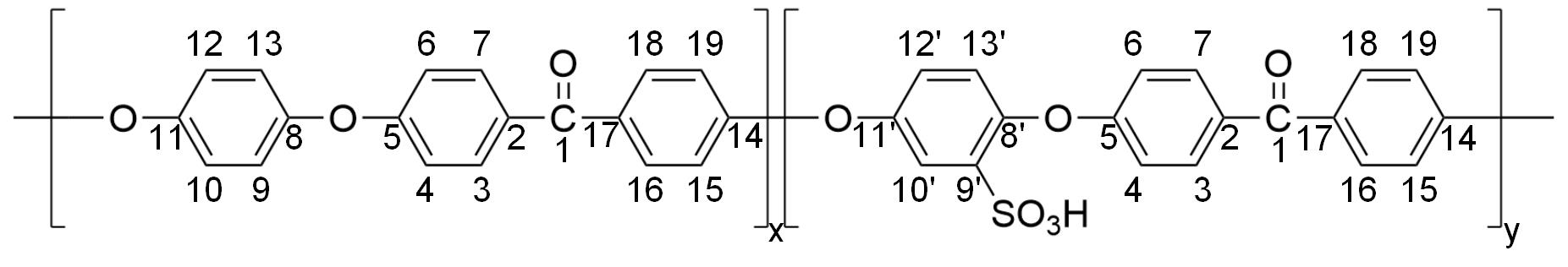


Figure 3. Structure and atom numbering of SPEEK.


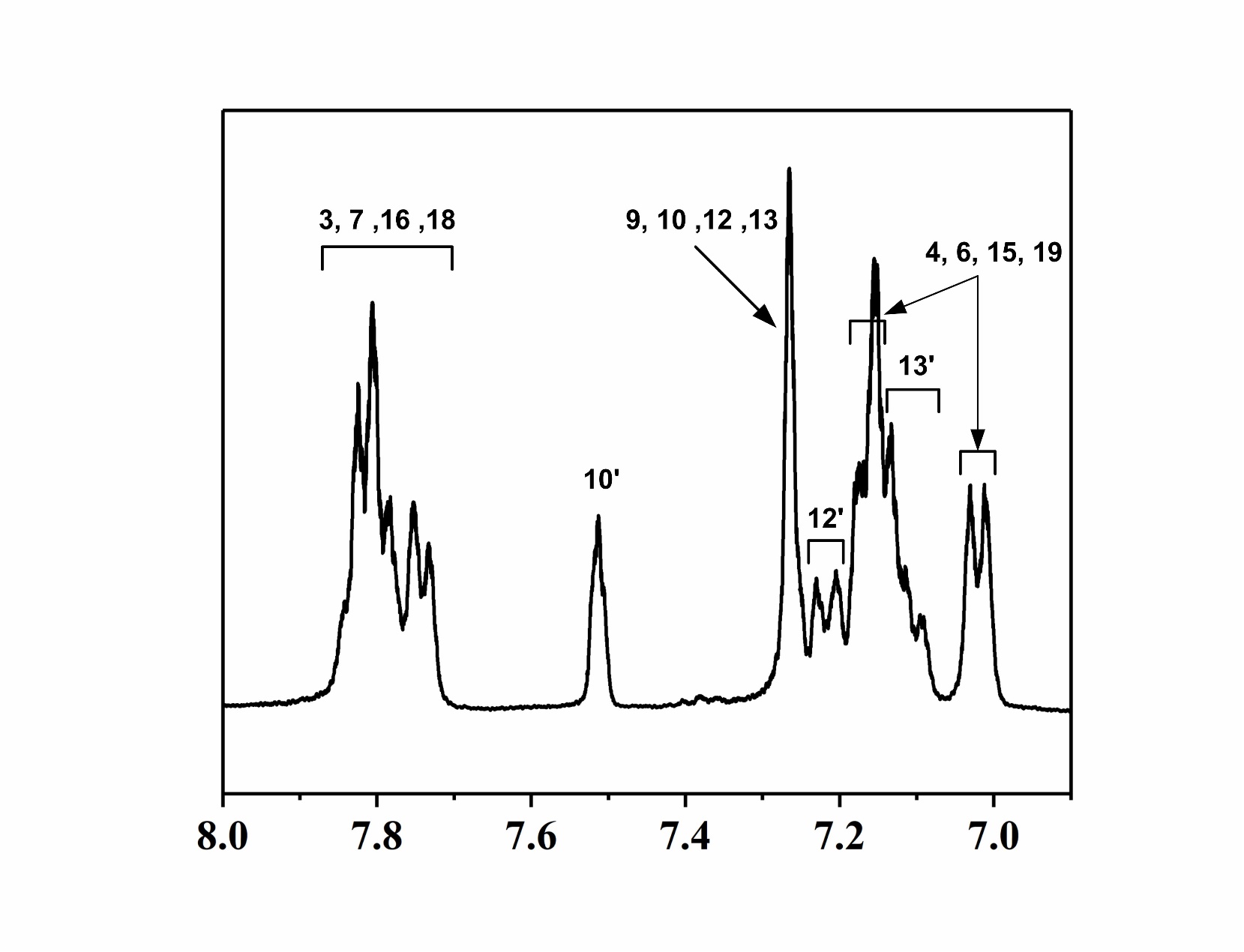


Figure 4. ^1^H NMR spectrum of the SPEEK membrane (M-3). From the spectrum, the degrees of sulfonation (DS) of the obtained SPEEK were calculated to be 62%. The specific peaks were associated with the corresponding H.

$$\frac{n}{12-2n}=\frac{A_{H_{10^{'}}}}{\sum H_{3,4,6,7,9,10,12,13,12^{'},13^{'},15,16,18,19}}$$

$$DS=n\times100\text{\%}$$

2.3 FT-IR measurements [1-2]


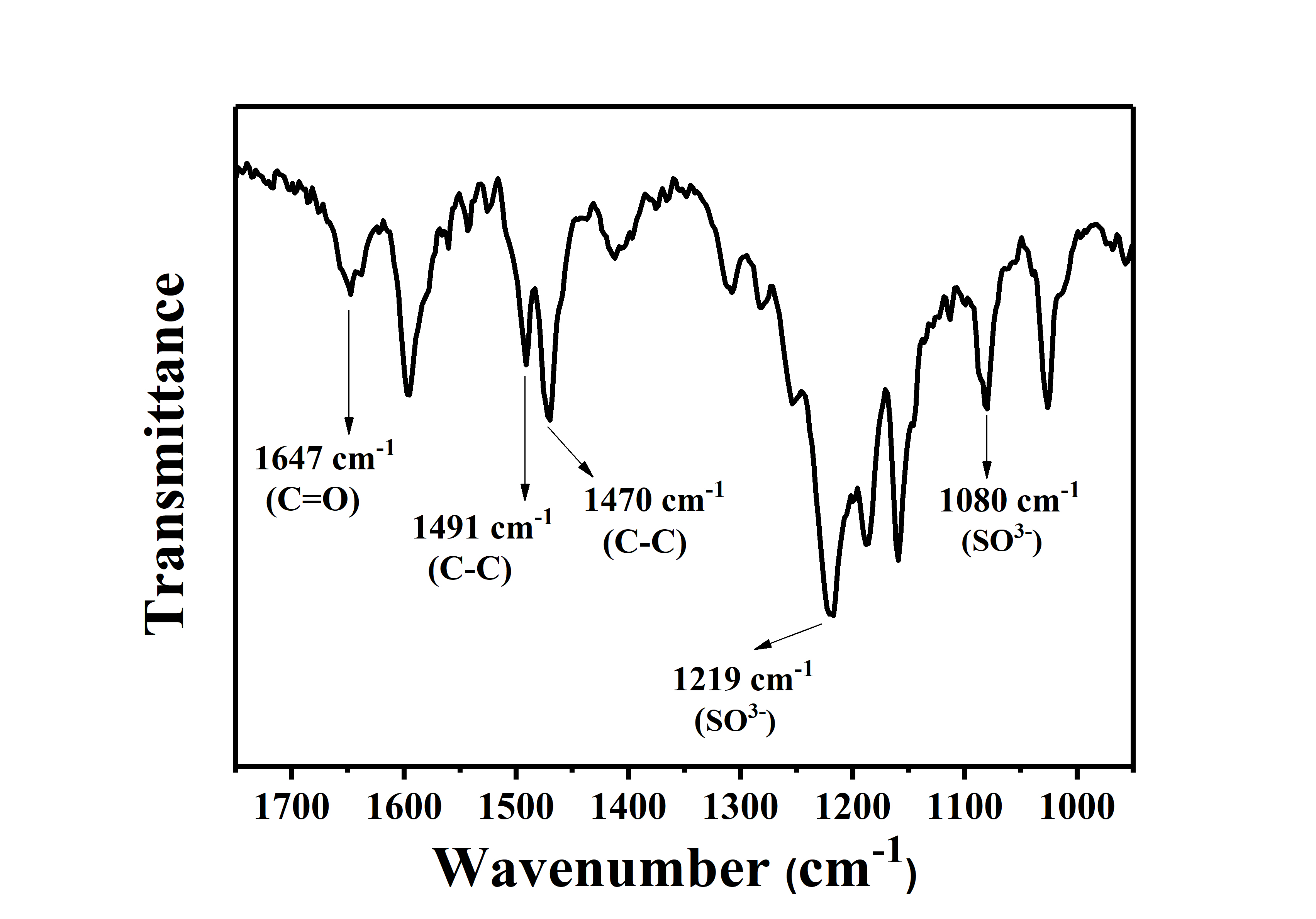


Figure 5. IR spectra of the SPEEK membrane. The absorption bands at 1080 cm^-1^ and in 1219 cm^-1^ are assigned to symmetric and asymmetric O=S=O vibration, respectively. The band appearing at 1647 cm^-1^ is attributed to C=O vibration. The absorption at 1470 cm^-1^ and 1491 cm^-1^ are concerned to the aromatic C−C band.

2.4 GPC measurements


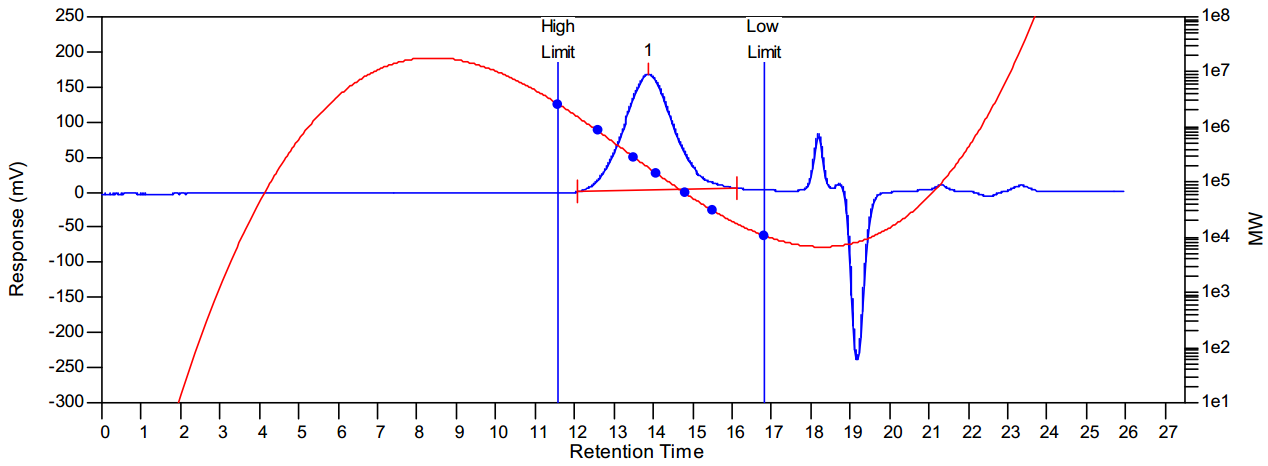


Figure 6. GPC curves of the SPEEK. ($M_{n}=142500$, $M_{w}=225200$, $PDI=1.58$)

2.5 AFM measurements

AFM views were recorded with Bruker (FASTSCANBIO).





Figure 7. AFM views of (a, c) skin layer surface and (b, d) supporting layer surface.

2.6 Contact angles measurements

A water drop with a volume of 2 *μ*L was dropped onto the membrane with a microsyringe in air. At least 3 contact angles at different locations for each membrane were recorded about 10 s after water drop contacting membrane. Ahead of the experiment, membranes were cut into the strip shape, fixed on the glass, and immersed them in deionized water for a period of time. After that, water on the membrane surface was removed by the filter paper carefully.





Figure 8. The optical images of water contact images of (a) skin layer and (b) supporting layer.

Section 3. Electrical Measurements [3]


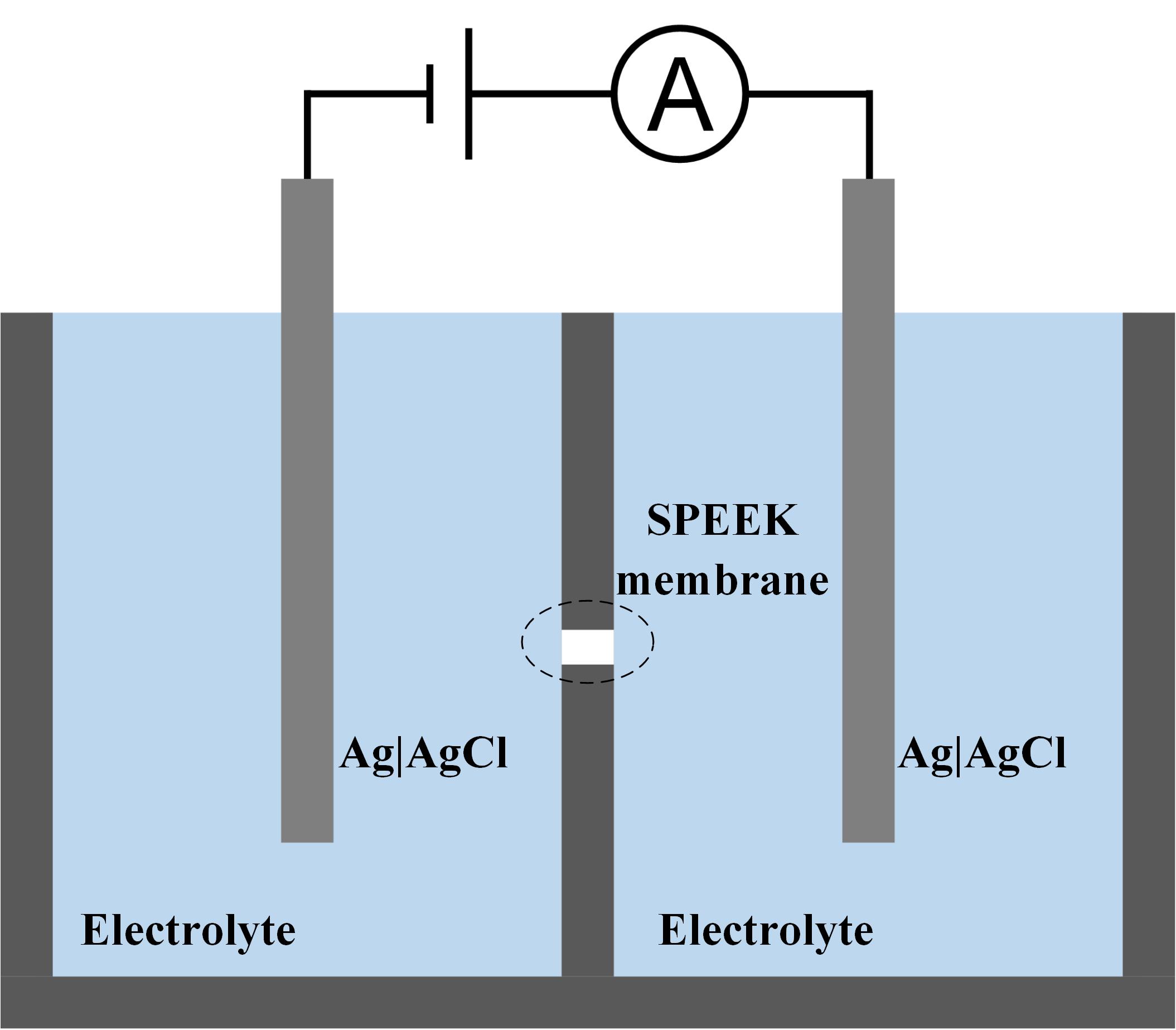


Figure 9. Schematic of the experimental setup.

Section 4. Ion selectivity of the membrane

4.1 The cation transference number

For SPEEK membrane, it behaves positive ion selection and the transference number $t_{\text{+}}$ is calculated following the equation [5]:

$$t_{\text{+}}=\frac{1}{2}\left(\frac{E_{\text{diff}}}{\frac{RT}{zF}\ln\frac{\gamma_{c_{\text{H}}}c_{\text{H}}}{\gamma_{c_{\text{L}}}c_{\text{L}}}}+1\right)$$

Where $t_{\text{+}}$ is the cation transference number; $E_{\text{diff}}$ refers to the diffusion potential; *R*, *T*, *z*, *F*, refer to the gas constant, temperature, valence charge and Faraday constant respectively; 𝛾 and 𝑐 refer to ion activity coefficient and concentration. As shown in Table 3, the transference number increases with decreasing concentration.

Section 5. Electrode calibration

The energy conversion performance measurement was conducted by testing the scanning *I-V* cycles under a concentration gradient. The sweeping voltages from -0.44 V to 0.44 V was applied with a step of 0.04 V. The intercept on the voltage axis ($V_{\text{OC}}$) is contributed by the redox potential ($E_{\text{redox}}$) on the electrode and the diffusion potential ($E_{\text{diff}}$) from the SPEEK membrane. The equivalent circuit of the testing system is as shown in Fig. 10. And their relationship can be displayed below [4]:

$E_{\text{diff}}$=$V_{\text{OC}}$−$E_{\text{redox}}$

The thermodynamic value of $E_{\text{redox}}$ can be calculated as:

$$E_{\text{redox}}=\frac{RT}{zF}\ln\frac{\gamma_{c_{\text{H}}}c_{\text{H}}}{\gamma_{c_{\text{L}}}c_{\text{L}}}$$

This method could largely preclude the influence bought by other unexpected factors [5], such as electrolyte imperfection or contamination etc. The obtained $V_{\text{OC}}$, $E_{\text{redox}}$, and $E_{\text{diff}}$ are shown in Table 2.


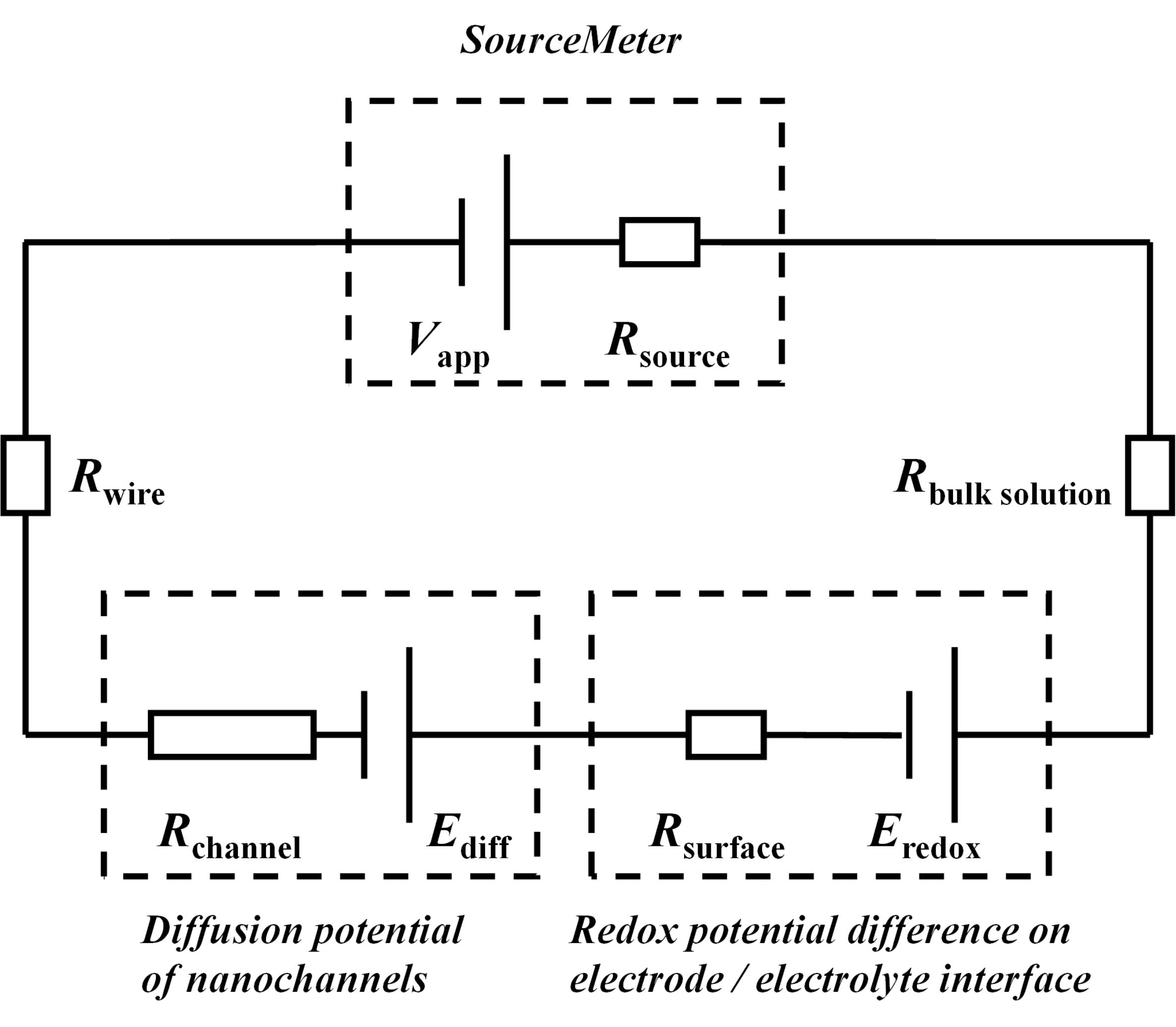


Figure 10. Equivalent circuit of the SPEEK membrane power source under a concentration gradient.

Table 2. The corresponding $V_{\text{OC}}$, $E_{\text{redox}}$, $E_{\text{diff}}$ value

| Concentration gradient (M/M) | 10^-5^/10^-4^ | 10^-5^/10^-3^ | 10^-5^/10^-2^ | 10^-5^/10^-1^ | 10^-5^/1 | 10^-5^/2 |
| --- | --- | --- | --- | --- | --- | --- |
| $V_{\text{OC}}$ (mV) | 93 | 160 | 233 | 271 | 344 | 332 |
| $E_{\text{redox}}$ (mV) | 59 | 117 | 174 | 230 | 282 | 299 |
| $E_{\text{diff}}$ (mV) | 34 | 43 | 58 | 40 | 61 | 33 |

Section 6. Energy Conversion Efficiency [6]

Energy conversion efficiency is defined as the ratio of the output energy (electrical energy) to the input energy (Gibbs free energy of mixing). For cation-selective system, maximum power generation, $\eta_{\max}$, can be calculated as:

$$\eta_{\max}=\frac{\left(2t_{\text{+}}-1\right)^{2}}{2}$$

The energy conversion efficiency under a series of concentration gradients can be calculated. As shown in Table 3, the efficiency increases with a decrease of concentration because the transference number increases. The best efficiency obtained is 16.7%.

Table 3. The cation transference number and the conversion efficiency of the SPEEK membrane at different salinity gradients. The asymmetric solutions are KCl solution.

| $c_{\text{H}}$ (M) | $c_{\text{L}}$ (M) | $E_{\text{diff}}$ (mV) | $t_{\text{+}}$ | *η* (%) |
| --- | --- | --- | --- | --- |
| 10^-4^ | 10^-5^ | 34 | 0.79 | 16.7 |
| 10^-3^ | 10^-5^ | 43 | 0.68 | 6.6 |
| 10^-2^ | 10^-5^ | 58 | 0.67 | 5.5 |
| 10^-1^ | 10^-5^ | 40 | 0.59 | 1.5 |
| 1 | 10^-5^ | 61 | 0.61 | 2.3 |
| 2 | 10^-5^ | 33 | 0.55 | 0.6 |

Section 7. The stability of generator in solutions at different pH


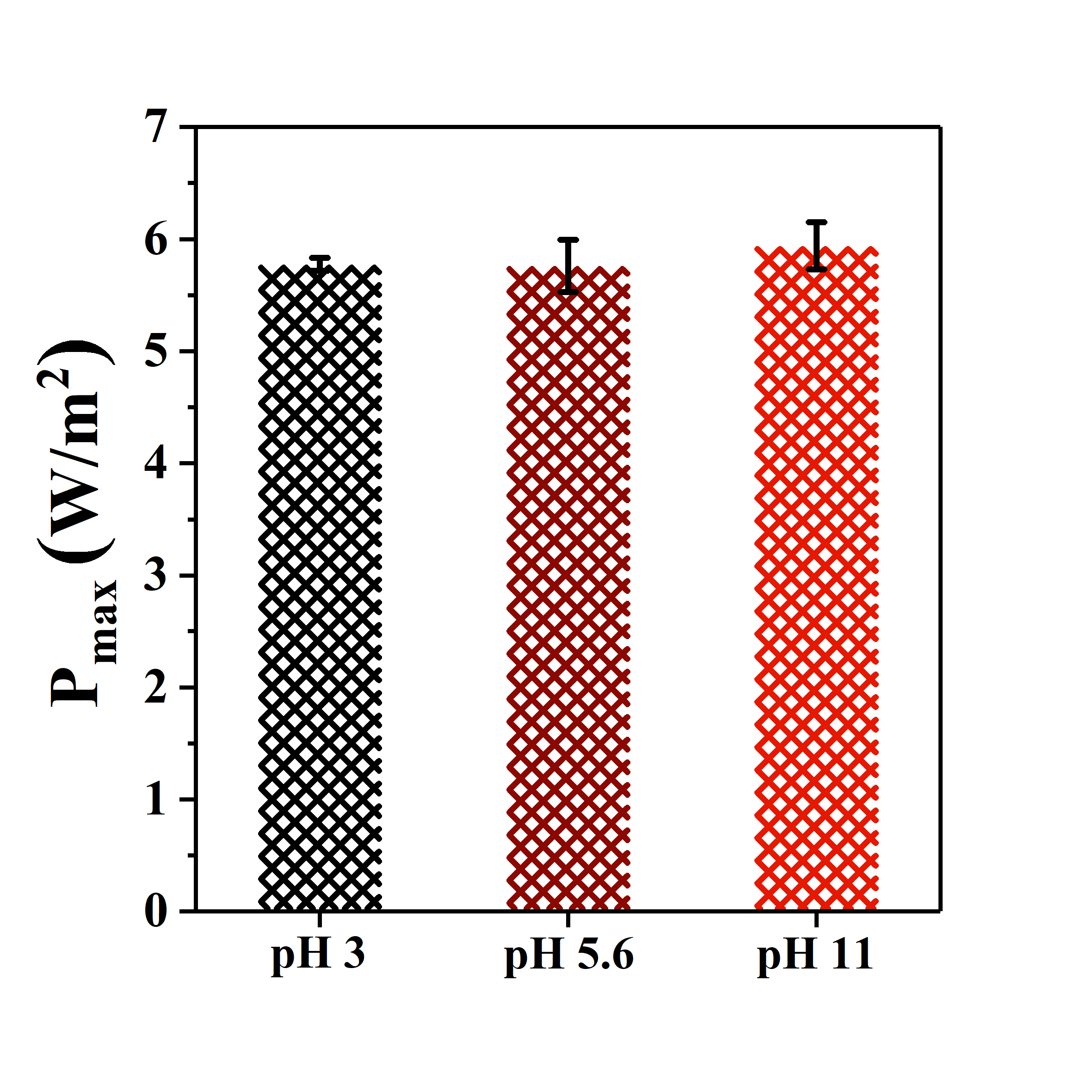


Figure 11. The power densities of the SPEEK-62% under solution at pH of 3, 5.6 and 11.

Section 8. Numerical simulation

8.1 Calculation of sapce charge density and surface charge density of ion channel

As shown in Fig. 12, nanochannel in SPEEK membrane’s skin layer was simplised as a 1000 nm long channel (diameter, 20-60 nm), in consistent with the experimental geometry values. There are two types of nanochannel which are investigated: space charge and surface charge. Bottom of Fig. 12 are the nanochannel and enlarged cross-section schematic of two kinds of channel.


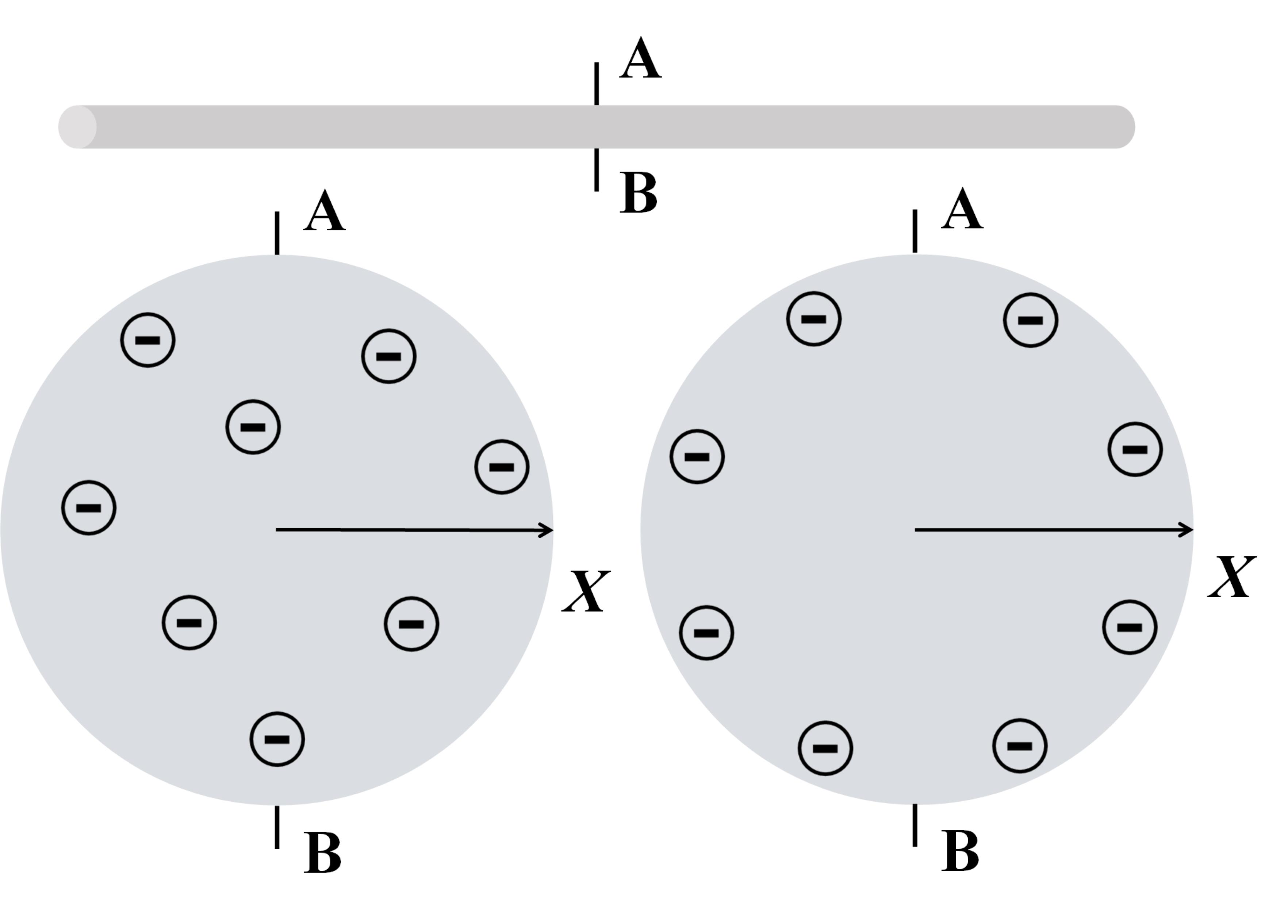


Figure 12. Illustration of two types of ion channel.

According to the relationship among the following symbols, as shown in Table 4, the space charge density and surface charge density could be roughly calculated as follwed.

Table 4. The symbols and definitions.

| *m_0_/m_1_/m_2_* | the mass of SPEEK membrane: total/skin layer/supporting layer |
| --- | --- |
| *A_1_/A_2_* | the area of SPEEK membrane’s cross section: skin layer/ supporting layer |
| *V_0_/V_1_/V_2_* | the volume of SPEEK membrane nased on cross section: skin layer/supporting layer |
| *ε_1_/ε_2_* | void ratio of dense skin layer/supporting layer |
| *F* | faraday constant |
| *M* | the molecular weight of SPEEK |
| *q_1_* | the charge of skin layer |
| *ρ* | the mass density of SPEEK membrane |
| *DS_1_/DS_2_/DS_3_* | the sulfonation degree of M-3/M-8/M-9 |
| $\overline{\sigma_{1}}$*/$\overline{\sigma_{2}}$/*$\overline{\sigma_{3}}$ | the average dense layer’s space charge of M-3/M-8/M-9 |
| $\sigma_{1}$*/$\sigma_{2}$/*$\sigma_{3}$ | the space charge of ion channal in M-3/M-8/M-9 |
| *Q_1_/ Q_2_* | the charge of individual ion channel with space charge/surface charge |
| $\delta$ | the surface charge density of ion channal |
| $V_{ion\mathop{}\limits channel}$ | the volume of individual ion channel with space charge |
| $A_{ion\mathop{}\limits channel}$ | the area of individual ion channel with surface charge |
| *L* | the length of individual ion channel |
| *d* | the diameter of individual ion channel |

$\frac{m_{1}}{m_{2}}=\frac{A_{1}\times\left(1-\varepsilon_{1}\right)}{A_{2}\times\left(1-\varepsilon_{2}\right)}$ (1)

$m_{0}=m_{1}+m_{2}$ (2)

$\frac{V_{1}}{V_{2}}=\frac{A_{2}}{A_{2}}$ (3)

$V_{0}=V_{1}+V_{2}$ (4)

$q_{1}=\frac{F\times m_{1}\times DS_{1}}{M}$ (5)

$\overline{\sigma}=\frac{q_{1}}{V_{1}}$ (6)

$V_{0}=\frac{m_{0}}{\rho}$ (7)

According to (1)(2)(5), obtain

$q_{1}=\frac{F\times DS}{M}\times m_{0}\times\frac{A_{1}\times\left(1-\varepsilon_{1}\right)}{A_{1}\times\left(1-\varepsilon_{1}\right)+A_{2}\times\left(1-\varepsilon_{2}\right)}$ (8)

According to (3)(4), obtain

$V_{1}=V_{0}\times\frac{A_{1}}{A_{1}+A_{2}}$ (9)

According to (6)(7)(8)(9), obtain

$\overline{\sigma_{1}}=\frac{F\times\rho\times DS}{M}\times\frac{\left(A_{1}+A_{2}\right)\times\left(1-\varepsilon_{1}\right)}{A_{1}\times\left(1-\varepsilon_{1}\right)+A_{2}\times\left(1-\varepsilon_{2}\right)}$ (10)

Assume that charged region is equal to uncharged region, obtain

$\sigma_{1}=2\times\overline{\sigma_{1}}$ (11)

In summary,

$\sigma_{1}=\frac{2\times F\times\rho\times DS}{M}\times\frac{A_{1}\times\left(1-\varepsilon_{1}\right)}{A_{1}\times\left(1-\varepsilon_{1}\right)+A_{2}\times\left(1-\varepsilon_{2}\right)}\times\frac{A_{1}+A_{2}}{A_{1}}$ (12)

$Q_{1}=\sigma_{1}\times V_{ion\mathop{}\limits channel}$ (13)

$V_{ion\mathop{}\limits channel}=L_{ion\mathop{}\limits channel}\times\pi\times\left(\frac{d}{2}\right)^{2}$ (14)

$Q_{2}=\delta\times A_{ion\mathop{}\limits channel}$ (15)

$A_{ion\mathop{}\limits channel}=L_{ion\mathop{}\limits channel}\times\pi\times d$ (16)

If the total charge of space charge nanochannel is eaqual to the total charge of surface charge nanochannel, $Q_{1}=Q_{2}$ (17)

In summary,

$\delta=\frac{\sigma\times d}{4}$ (18)

Since

$M=1.425\times10^{4}\frac{g}{mol}$, $F=96485\frac{C}{mol}$, $\frac{A_{1}}{A_{2}}=9.3$, $\rho=488.1\frac{kg}{m^{3}}$, $\varepsilon_{1}\sim0$, $\varepsilon_{2}\sim0.32$, $DS_{1}=0.62$, $DS_{2}=0.68$, $DS_{3}=0.72$

Obtain $\sigma_{1}=4.23\frac{C}{cm^{3}}$, $\delta=0.0423\frac{C}{\text{m}^{2}}$, $\sigma_{2}=4.64\frac{C}{cm^{3}}$, $\sigma_{3}=4.91\frac{C}{cm^{3}}$

8.2 Axial Symmetry [3, 7-8]

The inflence of space charge and surface charge in nanochannel is theoretically investigated using a commercial finite-element software package COMSOL Multiphysics. The coupled governing Poisson and Nernst-Planck equations are shown below:

, (i = +1 or −1)

where $\text{J}_{i}$, $D_{i}$, $c_{i}$, $z_{i}$, , $R$, $F$, and $T$refer to the ionic flux, diffusion coefficient which is $1\times10^{-9}$ $m^{2}\cdot s^{-1}$, ion concentration, valence number for each species i, electrical potential, universal gas constant, Faraday constant, and absolute temperature which is indeed room temperature, respectively. The relationship between the eletric potential and ion concentration satisfies the Poisson equation:

where $\varepsilon$ refer to dielectric constant of the electrolyte solutions, $\rho_{0}$ is the space charge density of the mobile ions ($\rho_{0}=F\sum_{i}z_{i}c_{i}$), $\rho_{s}$ is the space charge brought by SPEEK. When the system reaches a stationary regime, the ionic flux should satisfy the steady-state equation:

$$\nabla\cdot\text{J}_{i}=0$$

The couple equations are solved neglecting hydrodynamic effects and assuming appropriate boundary conditions. A sketch of the computation domain is shown in Figure S12. In order to gain affordable computation scale, the fluidic pathway through the composite membrane is simplified to be a 1000 nm long single channel (diameter, 40 nm). Two electrolyte reservoirs (400 × 200 nm) were added to minimize the influence of the resistance of mass transfer at the entrance and exit. The ion flux has the zero normal components at boundaries:

$$\text{n}\cdot\text{J}_{i}=0$$

In this work, the surface charge and space charge was set according to the calculation, within the proper ranges. The concentration of the electrolyte in the two reservoirs was set at 0.01 M.


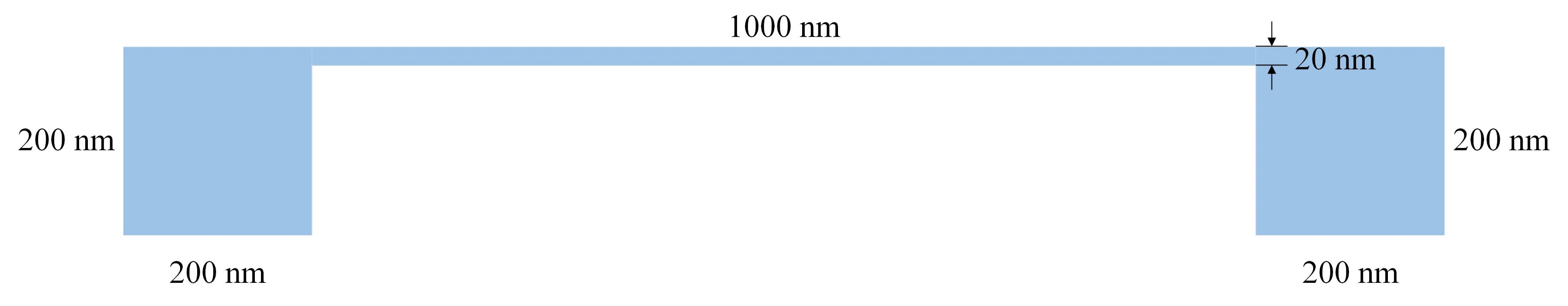


Figure 13. Model of theoretical simulation.





Figure 14. (a) Numerical simulation results of the K^+^ concentration distribution along the position (*X*) changing in space charged and surface charged nanochannels. The bulk KCl solution concentration is set at 0.01 M. (b) Integration of K^+^ concentration in nanochannel with space charge and surface charge when the KCl bulk solutions are set at 0.01 M, 0.1 M, 1 M, demonstrating that higher concentration of K^+^ exists in space charge channel which facilitates ion conductivity. Inset, the difference of K^+^ mole in space charged nanochannel and surface charged nanochannel. (c) Numerical simulation results of the K^+^ concentration distribution along the radial position (*X*) in space charges (σ_1_, σ_2_, and σ_3_) nanochannels, which are correspond to different DS (62%, 68%, 72%). (d) The calculated ionic concentration distribution in nanochannels with three space charges, confirming that high space charge can lead to high ionic concentration.

Section 9. Output power densities of different SPEEK membranes


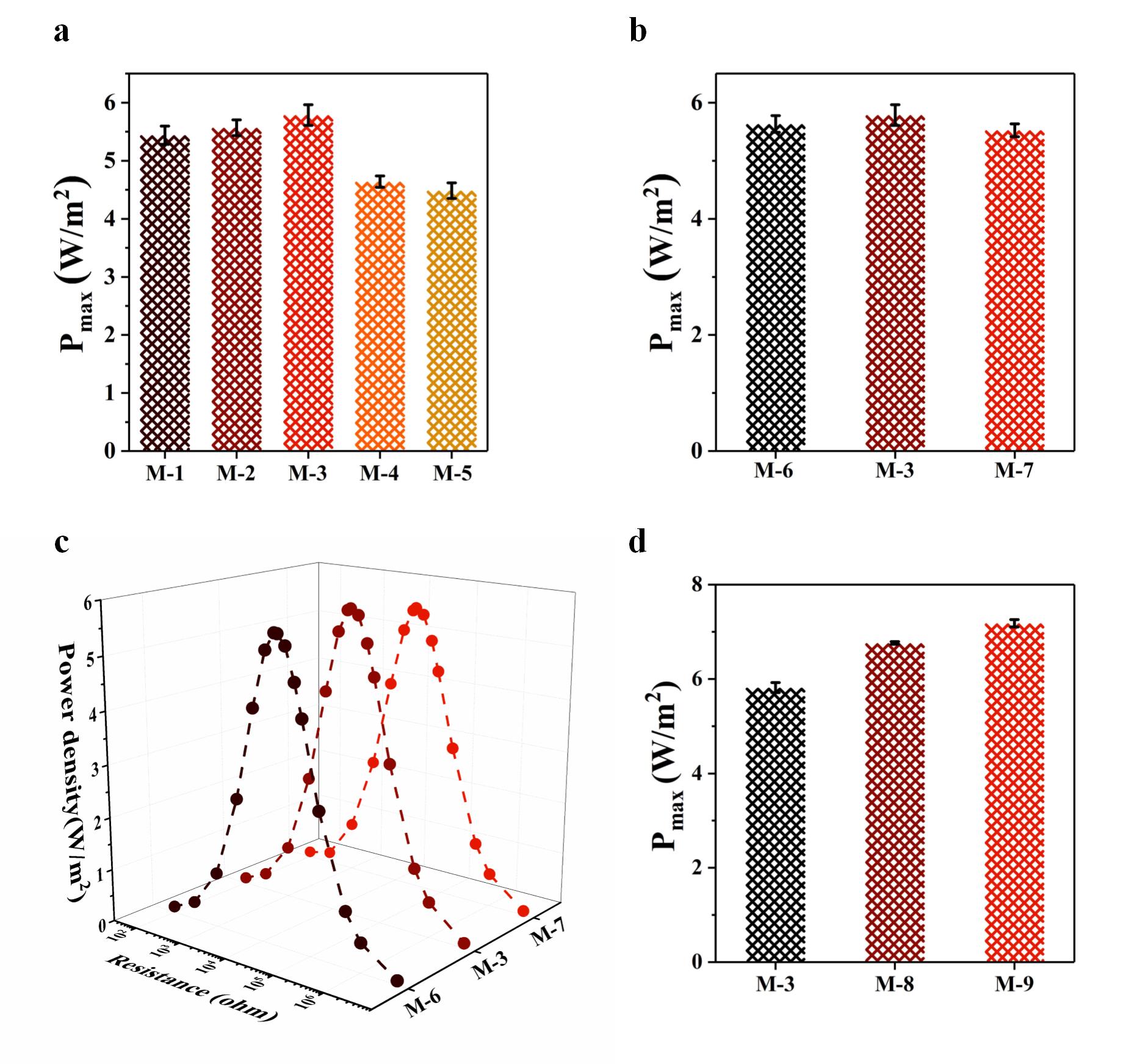


Figure 15. Osmotic energy conversion of the SPEEK membranes with different thickness (a), mass fraction (b and c) sulfonation degree (d).

Section 10. The thermal property


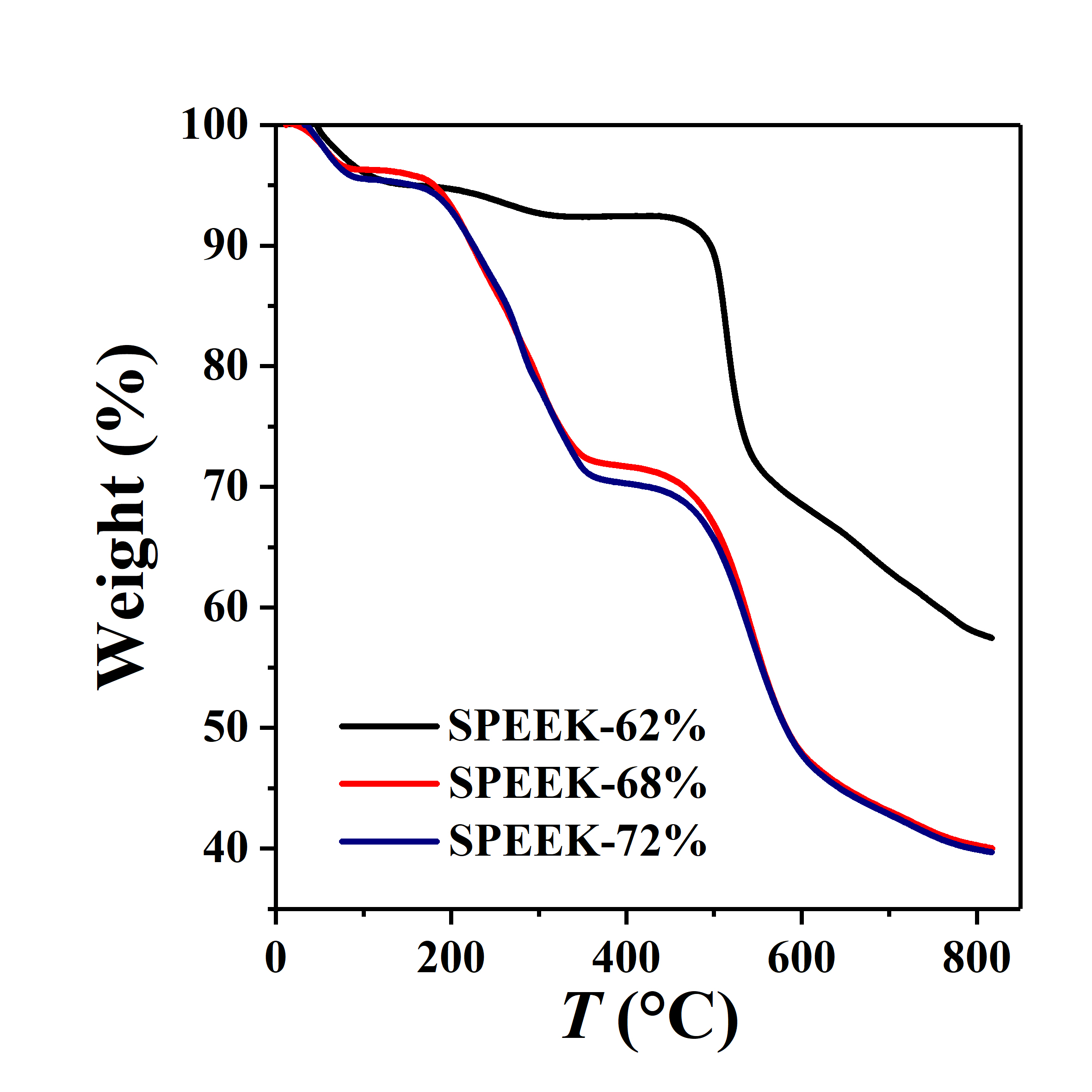


Figure 16. TGA curves of the SPEEK membranes with the DS of 62%, 68% and 72%.

1. Devaux J, Delimoy D and Daoust D *et al.* On the molecular weight determination of a poly(aryl-ether-ether-ketone) (PEEK). *Polymer* 1985; **26**: 1994-2000.

2. Xing P, Robertson GP and Guiver MD *et al.* Synthesis and characterization of sulfonated poly(ether ether ketone) for proton exchange membranes. *J. Membr. Sci.* 2004; **229**: 95-106.

3. Zhu X, Hao J and Bao B *et al.* Unique ion rectification in hypersaline environment: a high-performance and sustainable power generator system. *Sci. Adv.* 2018; **4**: eaau1665.

4. Feng J, Graf M and Liu K *et al.* Single-layer MoS_2_ nanopores as nanopower generators. *Nature* 2016; **536** (7615): 197-200.

5. Zhang Z, Sui X and Li P *et al.* Ultrathin and ion-selective janus membranes for high-performance osmotic energy conversion. *J. Am. Chem. Soc.* 2017; **139** (26): 8905-8914.

6. Kim D, Duan C and Chen Y *et al.* Power generation from concentration gradient by reverse electrodialysis in ion-selective nanochannels. *Microfluid. Nanofluid.* 2010; **9**: 1215-24.

7. Zhang Z, Yang S and Zhang P *et al.* Mechanically strong MXene/Kevlar nanofiber composite membranes as high-performance nanofluidic osmotic power generators. *Nat. Commun.* 2019; **10**: 2920.

8. White HS and Bund A. Ion current rectification at nanopores in glass membranes. *Langmuir* 2008; **24**: 2212-18.
